# OsNOA1 functions in a threshold-dependent manner to regulate chloroplast proteins in rice at lower temperatures

**DOI:** 10.1186/s12870-018-1258-9

**Published:** 2018-03-16

**Authors:** Han He, Qiaosong Yang, Boran Shen, Sheng Zhang, Xinxiang Peng

**Affiliations:** 1 State Key Laboratory for Conservation and Utilization of Subtropical Agro-bioresources, South China Agricultural University, Guangzhou, China; 2 Institute of Fruit Tree Research, Guangdong Academy of Agricultural Sciences, Guangzhou, China; 3 Institute of Biotechnology, Cornell University, Ithaca, USA

**Keywords:** Chloroplast, NOA1, Threshold-dependent, Quantitative proteomics, Rice, Tandem mass tag

## Abstract

**Background:**

Although decreased protein expressions have been observed in NOA1 (Nitric Oxide Associated protein 1) deficient plants, the molecular mechanisms of how NOA1 regulates protein metabolism remain poorly understood. In this study, we have used a global comparative proteomic approach for both *OsNOA1* suppression and overexpression transgenic lines under two different temperatures, in combination with physiological and biochemical analyses to explore the regulatory mechanisms of OsNOA1 in rice.

**Results:**

In *OsNOA1*-silenced or highly overexpressed rice, considerably different expression patterns of both chlorophyll and Rubisco as well as distinct phenotypes were observed between the growth temperatures at 22 °C and 30 °C. These observations led us to hypothesize there appears a narrow abundance threshold for OsNOA1 to function properly at lower temperatures, while higher temperatures seem to partially compensate for the changes of OsNOA1 abundance. Quantitative proteomic analyses revealed higher temperatures could restore 90% of the suppressed proteins to normal levels, whereas almost all of the remaining suppressed proteins were chloroplast ribosomal proteins. Additionally, our data showed 90% of the suppressed proteins in both types of transgenic plants at lower temperatures were located in the chloroplast, suggesting a primary effect of OsNOA1 on chloroplast proteins. Transcript analyses, along with in vitro pull-down experiments further demonstrated OsNOA1 is associated with the function of chloroplast ribosomes.

**Conclusions:**

Our results suggest OsNOA1 functions in a threshold-dependent manner for regulation of chloroplast proteins at lower temperatures, which may be mediated by interactions between OsNOA1 and chloroplast ribosomes.

**Electronic supplementary material:**

The online version of this article (10.1186/s12870-018-1258-9) contains supplementary material, which is available to authorized users.

## Background

NOA1 (Nitric Oxide Associated protein 1) was firstly reported as a NO synthase in *Arabidopsis* (AtNOS1) by Guo *et al*. [1]. However, subsequent studies failed to support the initial observation, thus the original nomenclature NOS1 was renamed to NO-associated protein 1 (NOA1) [2, 3]. In spite of this, extensive studies have shown that this protein is correlated in an indirect manner with NO production in plants [2–11]. Van Ree *et al*. [7] demonstrated that the decreased NO was caused by the shortage of photosynthates due to chloroplast defects in the *NOA1* mutant. Recently, Kwan *et al*. [12] further confirmed AtNOA1 was not an authentic NOS. Homologs of NOA1 have been identified in both bacteria and human, which were named as YqeH and C4orf14, respectively. Further analyses showed these equivalent proteins are linked to the assembly of small ribosomal subunits in bacteria and to the earliest stages of 28S subunit assembly in mitochondria of human cells, respectively [2, 13–15]. In animals, NOA1 homologs were reported to target to mitochondria [14, 15], while plant homologs were known to be localized in chloroplast [6, 11, 16–18]. Yellowish phenotypes have been observed in either *Arabidopsis* or rice mutant which is deficient in the *NOA1* gene [2, 6, 16, 17]. Thus the biological role of plant NOA1 is expected to be primarily associated with chloroplast ribosome functions, and has been extensively studied in recent years by several groups. Liu *et al*. [16] have reported the proteome changes in the rice RNAi mutant compared to WT in support of the expected functional role of OsNOA1 in chloroplast translation. Qi *et al*. [18] reported NOA1 was required for thylakoid protein complex assembly and functionally related with VAR2, further suggested its’ role in chloroplast ribosome assembly and translation. Our previous study showed that when *NOA1* was suppressed the transgenic plants were temperature-sensitive, in which lower temperatures aggravated the deficiency of chlorophyll and Rubisco while a higher temperature could help maintain normal phenotypes. Additionally, this regulation occurred at the level of protein biosynthesis [17]. Our previous findings on the NOA1’s functioning in a temperature-dependent manner for regulating chlorophyll biosynthesis and Rubisco formation, is of particular significance, since further study on the effect of NOA1 deficiency under variable temperatures on global protein expression and on chloroplast ribosome functions could shed light on the role of NOA1 and its regulation network in plants, particularly in regard to temperature adaptation or tolerance mechanism in plants.

While our earlier microarray analysis identified a number of possible candidate genes, which may be involved in OsNOA1 function [17], a complementary study of the rice proteome profiles under the same condition has the potential to yield new insights into the biological function of NOA1 and molecular mechanism of NOA1 associated regulation. In this study, we utilized previously-generated RNAi and newly-generated overexpression lines to further investigate the function of NOA1 in rice. Here we report the proteome changes which include important candidate proteins involved in chloroplast ribosome biogenesis and several classes of proteins with significant changes in response to various levels of OsNOA1 under two different temperature conditions. Our results suggest that OsNOA1 can function in a threshold-dependent manner to regulate the biosynthesis of chloroplast proteins in rice at lower temperatures, and this regulation may be achieved via interactions between NOA1 and chloroplast ribosomes.

## Methods

### Chemicals and materials

Sequence-grade acetonitrile (ACN), trifluoroacetic acid (TFA), and formic acid (FA) were purchased from Fisher Scientific (Fair Lawn, NJ). The tandem mass tag (TMT) 6-plex kit was purchased from Thermo Scientific (Rockford, IL) and SCX cartridges were purchased from AB Sciex (Foster City, CA), the Sep-Pak solid-phase extraction (SPE) cartridges from Waters (Milford, MA), and modified trypsin from Promega (Madison, WI). The rest of the chemical reagents, unless otherwise noted, were obtained from Aldrich (Milwaukee, WI).

### Plant materials and growth conditions

The seeds of rice (*Oryza sativa*) cv. Zhonghua 11 (japonica cultivar-group, provided by the state key laboratory for conservation and utilization of subtropical agro-bioresources) were used for the construction of transgenic lines. Zhonghua 11, together with previously generated *OsNOA1*-silenced line (RNAi-20) [17] and newly constructed *OsNOA1* overexpression lines (Ox-2, Ox-22, Ox-45) were used for the physiological study and subsequent quantitative proteomic analyses. Pre-germinated seeds were grown in Kimura B complete nutrient solution, which was renewed every 3 days in a phytotron chamber, photosynthetically active radiation 80 μmol•m^− 2^•s^− 1^, relative humidity 65%, photoperiod 12 h light/12 h dark, 22/22 °C or 30/30 °C (light/dark).

### Construction of *OsNOA1*-overexpressed lines

To generate the *OsNOA1* overexpression construct, the cDNA of *OsNOA1* (1.7 kb) was amplified by RT-PCR with a forward primer 5′- TATGGTACCTCCTCCTGCTCCTAGT-3′ and a reverse primer 5′- CAGACGCGTGCTCTATTTGGACTAC-3′ from *Oryza sativa* (japonica cultivar-group). It was inserted at the MCS between *Kpn* I and *Mlu* I in the binary vector pOx which was kindly provided by Dr. Yao-Guang Liu from College of Life Sciences, South China Agricultural University, China. DNA sequencing confirmed the correct orientation and the cDNA identity [100% identical to *Os02g0104700* reported in NCBI]. The constructed vector named pOx-Ubi-OsNOA1 was then transformed into rice callus via *Agrobacterium* mediated infection (strain EHA105). Southern blot acquired the single-copy transgenic rice, and further screening for hygromycin resistance identified the homozygous lines.

### Expression and purification of recombinant proteins

For the expression of OsNOA1 in *E. coli*, the cDNA encoding OsNOA1 was generated by RT-PCR and cloned into the pCold I vector (Takara, Japan). Cell growth and protein induction were performed as previously reported [19]. Briefly, cells were cultured at 37 °C until the OD_600_ reached 0.4–0.6, and 0.1 mM IPTG was added to induce protein expression. After 24 h of induction at 16 °C, cells were harvested and lysed in lysis buffer (50 mM sodium phosphate, pH 7.8, 5% glycerol), then centrifuged for 15 min at 12000 rpm, 4 °C. The supernatant was collected and target protein was purified according to the Profinity™ IMAC protocol. Finally, purified protein was desalted and concentrated by ultrafiltration.

### Measurement of Rubisco abundance and western blot analyses

Leaf proteins were extracted by homogenizing 0.1 g of fresh leaves in 0.5 mL of 20 mM phosphate buffer (pH 7.5). The homogenate was centrifuged at 12,000×g for 15 min. Total amount of soluble proteins was determined by the Bradford method using BSA as a standard [20]. The content of Rubisco was measured by extraction of Coomassie brilliant blue R-250-stained subunit bands from SDS-PAGE gels with BSA as a standard [21].

For Western blot analysis of OsNOA1, leaf proteins from equal fresh weights were loaded in each lane, separated by 12.5% SDS-PAGE, transferred onto a nitrocellulose membrane, finally immunodetected with rabbit anti-OsNOA1 serum (made in-house) at 1:1000 dilutions. Goat anti-rabbit IgG (1:10,000) conjugated with horseradish peroxidase was used as the secondary antibody and the bands were detected with Amersham ECL Plus kit.

### Measurement of chlorophyll content

Chlorophyll was extracted from leaves with mixed solution (45% acetone, 45% ethanol and 10% distilled water), the content was determined spectrophotometrically at 645 nm and 663 nm [22].

### Quantitative and semi-quantitative RT-PCR

Total RNA was isolated using TRIZOL reagents (Invitrogen). One microgram of RNA was used as a template for the first-strand cDNA synthesis using ReverTra Ace (Toyobo, Japan) with random hexamers primers according to the manufacturer’s instructions. The quantitative RT-PCR reaction consisted of 10 μL 2 × SYBR Green PCR Master Mix (Toyobo), 200 nM primers, and 2 μL of 1:10-diluted template cDNA in a total volume of 20 μL. *OsNOA1* was quantified with a forward primer 5’-GGCGTCACTGGGGTTATATC-3′ and a reverse primer 5’-GCCATCGTCCTTAGCATAGC-3′. Transcript levels were measured by the DNA Engine Option 2 Real-Time PCR Detection system and processed by the Opticon Monitor software (Bio-Rad, USA). The data was normalized to the *OsActin1* gene (*Os03g0718100*).

The semi-quantitative RT-PCR reaction consisted of 10 μL 2 × Taq Master Mix (Sangon, Shanghai), 200 nM primers and 2 μL of 1:10-diluted template cDNA in a total volume of 20 μL. Primers used for amplifying rRNA were shown in Table 1. The semi-quantitative RT-PCR experiments were repeated with cDNA from three different batches.

**Table 1:** Primers used for quantifying rRNA

Primer IDs	Sequence (5′-3′)
OsActin1-F	GACATTCAGCGTTCCAGCCATGTAT
OsActin1-R	TGGAGCTTCCATGCCGATGAGAGAA
16S rRNA-F	CGGTATCTGAGGAATAAGCATCGGC
16S rRNA-R	TTGCTCCCCTAGCTTTCGTCTCTCA
16S rRNA precusor-F	AGGGTTGGCTATACTGCTGGTG
16S rRNA precusor-R	CCTGGGATTTGACGGCGGACT
23S rRNA-F	AATCAGCGGATGAGTTGTGGTTAGG
23S rRNA-R	CTCCTTTATCACTGAGCGGTCATTT
23S rRNA precusor-F	CGCATTCATGGACGTTGATAAGA
23S rRNA precusor-R	CCCTTAACCAAGCCACTGCCTAT

### Preparation of protein samples for proteomics research

Two batches of rice seedlings (set1 and set2) were cultured for the proteomics analysis; three biological replicates were separately sampled in each set, and each biological replicate contained 0.1 g fresh weight of rice leaves which came from 30 seedlings (leaves were cut into fine pieces and mixed, and then 0.1 g was weighed). All these samples were stored at − 80 °C for later protein sample preparation. Protein samples were obtained as follows: three biological replicates from each set were pooled together, ground in liquid N_2_ and fully homogenized with ice-cold sodium phosphate buffer (100 mM, pH 7.5) containing 1 mM EDTA, 100 μg/mL PMSF and 0.1% Triton X-100. The resulting homogenate was centrifuged at 12,000×g for 15 min and supernatant was collected. The supernatant proteins were lyophilized and stored at − 80 °C for further analysis.

### Proteomic profiling using TMT labeling and LC/MS-MS

TMT labeling was conducted according to protocols reported previously [23]. Briefly, protein samples were first denatured by adding 2% SDS and reduced with 50 mM Tris- (2-carboxyethyl) phosphine (TCEP) at 56 °C, Cysteine residues were alkylated with 20 mM iodoacetamide in the dark and quenched by addition of 20 mM dithiothreitol (DTT). The proteins were then digested with trypsin at 36 °C overnight, and subjected to TMT labeling. The digested peptides were labeled with TMT 6-plex reagents, the following 6 samples: WT, RNAi-20, Ox-2, Ox-45 grown at 22 °C, and WT and RNAi-20 grown at 30 °C were labeled respectively with 126-tag, 127-tag, 128-tag, 129-tag, 130-tag and 131-tag. Efficiency of TMT 6-plex labeling was assessed by MALDI-TOF/TOF 4700 (AB Sciex) by choosing 5 peptides from each sample randomly.

After labeling, the six samples were combined and subjected to high pH reverse phase (hpRP) fractionation. The pooled TMT labeled peptides were run through SCX cartridges and desalted by Sep-Pak SPE cartridges for subsequent hpRP separation. The hpRP chromatography was carried out using a Dionex UltiMate 3000 high performance LC system with a built-in microfraction collection option in its autosampler and UV detection (Thermo-Dionex, Sunnyvale, CA). The TMT-tagged tryptic peptides were reconstituted in buffer A (20 mM ammonium formate, pH 9.5, in water) and loaded onto an XTerra MS C_18_ column (3.5 μm, 2.1 × 150 mm, Waters, Milford, MA) with 20 mM ammonium formate (NH_4_HCO_2_, pH 9.5) as buffer A and 80% ACN/20% 20 mM NH_4_HCO_2_ as buffer B [24]. Forty-eight fractions were collected and pooled into final 12 fractions [25]. The fractions were dried and reconstituted in 2% ACN/0.5% FA for nano LC-MS/MS analysis.

Each fraction (5 μL) acquired above was then analyzed with a LTQ-Orbitrap Velos (Thermo-Fisher Scientific, San Jose, CA) mass spectrometer equipped with a “CorConneX” nano-ion source (CorSolutions LLC, Ithaca, NY), containing a PepMap C_18_ RP nano column (3 μm, 75 μm × 15 cm, Dionex) connected to a 10 μm analyte emitter (NewObjective, Woburn, MA) in duplicates and named as set1 and set2 [23]. Raw data sets were acquired with Xcalibur 2.1 software (Thermo-Fisher Scientific), and made publicly available at PRIDE database (http://www.ebi.ac.uk/pride/archive/projects/PXD005841, accession: PXD005841, Username: reviewer20210@ebi.ac.uk; Password: VhUtq7lM) [26].

### Protein identification and quantitation

Raw data acquired from the Orbitrap were converted into MGF files using Proteome Discoverer 1.2 (PD 1.2, Thermo). Subsequent database searches were carried out by Mascot Daemon (Version 2.3, Matrix Science, Boston, MA) for both protein identifications and TMT 6-plex quantitation against rice reference protein database downloaded from NCBI. The rice protein database is a protein preferred database containing the combined proteins predicted by both longest six frame translations and expressed sequence tag (EST) scan. The default Mascot search settings used for 6-plex TMT quantitative processing and protein identification were as follows: one missed cleavage site by trypsin allowed with fixed carbamidomethyl modification of cysteine, fixed 6-plex TMT modifications on Lys and N-terminal amines and variable modifications of methionine oxidation, deamidation of Asn and Gln residues. The peptide and fragment mass tolerance values were 20 ppm and 0.1 Da, respectively.

To estimate the false discovery rate (FDR) for a measure of identification certainty in each replicate set, we employed the target-decoy strategy of Elias and Gygi [27]. Specifically, an automatic decoy database search was performed in Mascot by choosing the decoy checkbox in which a random sequence of the database is generated and searched for raw spectra along with the real database. To reduce the probability of false peptide identification, only peptides with significance scores greater than “identity” at the 99% confidence interval by a Mascot probability analysis were counted as identified. It was required that each confident protein identification be based on at least two unique peptide identifications shown in Mascot. Proteins identified within the same family were grouped in the Mascot protein family summary. Furthermore, to be considered confidently quantified, we required that a protein produced at least two unique peptides which generate a complete TMT reporter ion series. The quantitative protein ratios were obtained by weighted quantitative peptides using 126-tag (for WT at 22 °C) or 130-tag (for WT at 30 °C) respectively as a denominator. The final ratios for each of the identified proteins were normalized using the median ratio with outlier removal setting automatic in Mascot for each set of experiments. The manufacturer’s recommended isotope correction factors were applied.

The threshold of significance was determined by fitting the data to 50 different theoretical distributions using EasyFit (MathWave Technologies, http://www.mathwave.com). The fits to each of the distributions was ranked using the Kolmogorov-Smirnov, Anderson-Darling and Chi-squarred tests. The standard deviation of the top ranked fit was used to estimate the fold change threshold of 95% confidence (2σ). Furthermore, a T-test was used to determine if the mean of the biological replicates of the “mutant samples” were statistically different from that of the WT controls. All protein species whose apparent expression ratios exceeded the thresholds of significance (±2-fold) and exhibited a *p*-value of ≤0.05 were reported as significantly changed.

### Functional classification and prediction of interaction proteins with NOA1

The functional annotation and classification of all identified proteins with a differential expression of over ±2-fold was determined according to Blast2go (Bioinformatics Department, CIPF, Valencia, Spain) [28]. Functional interaction network of proteins with OsNOA1 was predicted using EMBL STRING 10.0 web-based software (http://string-db.org/) [29], which consisted of either known or predicted interactions.

### His-tag pull-down assay

His-tag pull-down assays were done according to Zhang *et al*. [30], 100 μl (0.5 μg•μl^− 1^) of N-His-OsNOA1 was mixed with equal volume of 2 × binding buffer (50 mM PBS, 300 mM NaCl, 20 mM imidazole) and incubated with 1 ml of pre-equilibrated Ni-IDA resin at 4 °C for 30 min. Then 5 mg of crude protein extracted from rice leaves was added to the solution and incubated at 4 °C for 1 h. After that, the resin was washed with 10 ml 1 × binding buffer (50 mM PBS, 150 mM NaCl, 10 mM imidazole). Finally, OsNOA1 and its interaction proteins were eluted with 2 ml of 50 mM PBS (pH 8.0) containing 150 mM NaCl and 250 mM imidazole, The interaction proteins of OsNOA1 were separated by SDS-PAGE and identified by LC-MS/MS performed by BGI (BGI-Shenzhen, China).

## Results

### *NOA1*-overexpressed lines showed similar phenotypes to the *NOA1*-silenced lines

**Fig. 1:**
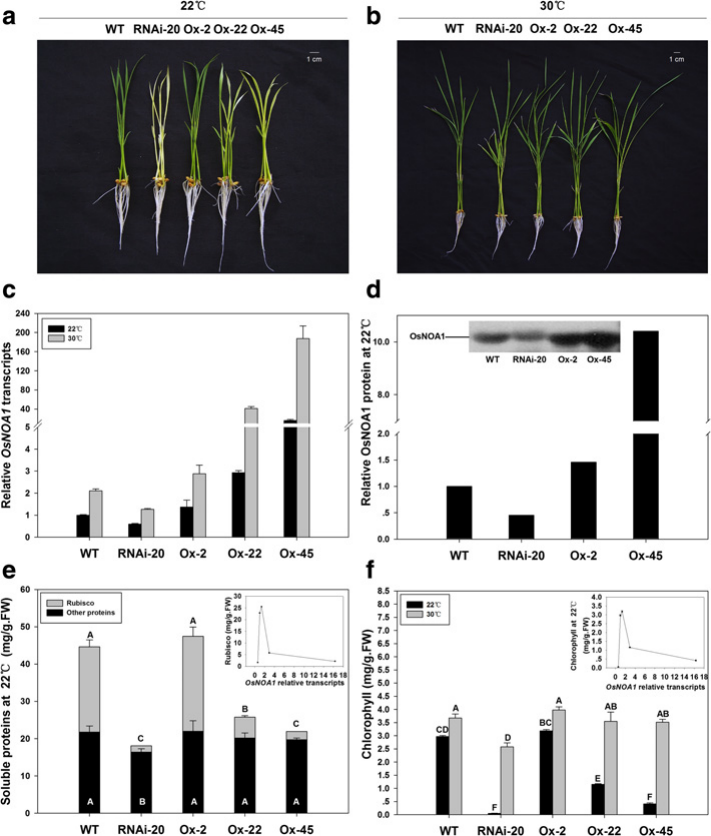
Phenotypes of transgenic plants (RNAi-20, Ox-2, Ox-22 and Ox-45) along with WT grown at different temperatures. Germinated seeds were grown in growth chambers at 22 °C (**a**) or 30 °C (**b**) under 80 μmol•m^− 2^ s^− 1^ light intensity. Transcripts of *OsNOA1* were determined by real-time PCR (*n* = 4) under both temperatures (**c**), and the mean values of NOA1 protein in two biological sets grown at 22 °C were detected by TMT-LC-MS/MS (**d**). Contents of soluble proteins (**e**) and chlorophyll (**f**) were determined (n = 4). Plots of abundance of Rubisco and chlorophyll against *OsNOA1* expression level were shown in the inset of (**e** and **f**), respectively. Data are means ±SD of n biological replicates and representative of three independent experiments. For (**e** and **f**), data significances were determined by DMRT (Duncan’s new Multiple Range Test). Differences were considered significant at the level of *P* < 0.01

We have previously obtained the *OsNOA1*-silenced lines and found these transgenic plants displayed temperature-sensitive yellowish phenotypes; i. e. a lower temperature aggravated the deficiency of chlorophyll and Rubisco while a higher temperature could help maintain normal phenotypes like WT [17]. In this study, we further generated the *OsNOA1*-overexpression lines (Ox lines). Three *OsNOA1*-overexpression lines (i. e. Ox-2, Ox-22 and Ox-45) together with the silenced line (RNAi-20) constructed previously were used for a comparative study. Interestingly, some Ox lines exhibited very similar phenotypes as compared to the silenced line (Fig. 1a, b) [17]. Investigation of both the mRNA and protein levels by quantitative RT-PCR and Western blot confirmed overexpression of the *OsNOA1* gene indeed occurred in those Ox lines (Fig. 1c, d), indicating the effect of overexpression is responsible for the similar phenotypes like the silenced line. Additionally, as the levels of *OsNOA1* increased in different Ox lines, the chlorosis became increasingly severe at lower temperatures (Fig. 1a), while normal green leaves were developed at a higher temperature for all lines (Fig. 1b–d). Further quantitative RT-PCR analysis revealed that as the extent of *OsNOA1* overexpression was increased both chlorophyll and Rubisco levels were slightly increased at first, and then decreased rapidly (see the insets in Fig. 1e, f).

These observations strongly suggested there is an abundance threshold for OsNOA1 to affect chlorophyll and Rubisco. It was also noticed that the total amount of other soluble proteins (except for Rubisco) was little affected in both silenced and Ox lines at the lower temperature (22 °C) compared to WT (Fig. 1e), revealing a primary effect of OsNOA1 on the metabolism of chloroplast proteins.

### Decrease of Rubisco abundance occurred at its biosynthetic level

**Fig. 2:**
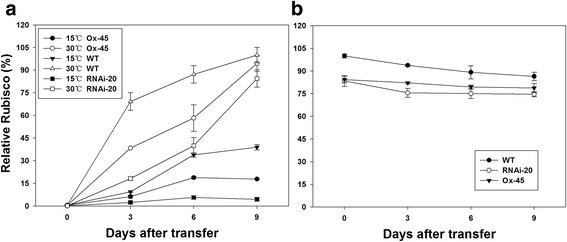
Changes of Rubisco abundance in etiolated seedlings after being exposure to light (**a**) or in non-etiolated seedlings after being transferred to the lower temperature (**b**). A: Germinated seeds were first grown in a dark chamber under 30 °C for 4d, the etiolated seedlings were then transferred to either 30 °C or 15 °C for various times (3d, 6d and 9d). The first fully expanded leaves were collected at 0d and leaves at the same leaf-position were sampled at other time points for analyses (n = 4); B: the post-germinated seeds were first grown at 30 °C for 10d, then transferred to 15 °C for various time (3d, 6d and 9d). The first fully expanded leaves were collected at 0d and leaves at the same leaf-position were sampled at other time points for analyses (n = 4). Data are means ±SD of n biological replicates and representative of three independent experiments

Rubisco is the most abundant chloroplast protein encoded by both the nucleus and chloroplast genomes and accounts for more than 50% of total soluble proteins in plant leaves [31]. So Rubisco was an ideal marker protein to check what had happened in the chloroplasts of the Ox lines. As shown in Fig. 2a, etiolated WT seedlings could restore Rubisco accumulation readily once return to light, at either 30 °C or 15 °C. In contrast, the etiolated Ox seedlings accumulated Rubisco to normal levels at 30 °C, but lost this ability at 15 °C. On the other hand, non-etiolated Ox seedlings as well as WT seedlings could maintain the Rubisco levels for various times, even after being shifted to 15 °C (Fig. 2b). In this regard, the Ox lines showed highly similar results (Fig. 1) to the silenced line as we reported before [17], further suggesting NOA1 regulates the abundance of chloroplast proteins at their biosynthetic levels.

### Chloroplast 16 s rRNA precursor highly accumulated in suppressed line of *OsNOA1*

**Fig. 3:**
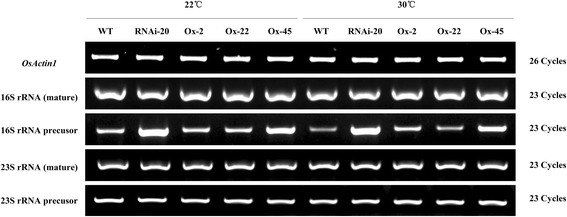
Semi-quantitative RT-PCR analyses of chloroplast rRNAs. Semi-quantitative RT-PCR analyses were performed using plants grown at either 22 °C/22 °C or 30 °C/30 °C (day/night). Primers used for RT-PCR were listed in Table 1

As summarized by Bharat [32], mutants which have defects in ribosome assembly or rRNA processing exhibited three common phenotypes: 1) accumulation of rRNA precursors; 2) cold-sensitive; 3) changes in the distribution of the ribosomal components. Therefore, we performed a semi-quantitative RT-PCR to examine the contents of chloroplast rRNAs. In Fig. 3, the suppressed line and the highly overexpressed line Ox-45 accumulated a great deal of 16S rRNA precursor, whereas other rRNAs were not notably changed including the mature form of 16S rRNA (Fig. 3). This result implicates OsNOA1 is essential for the normal function of chloroplast 30S ribosome.

### Proteomics profiling of WT, *NOA1*-silenced and Ox lines upon temperature changes

**Fig. 4:**
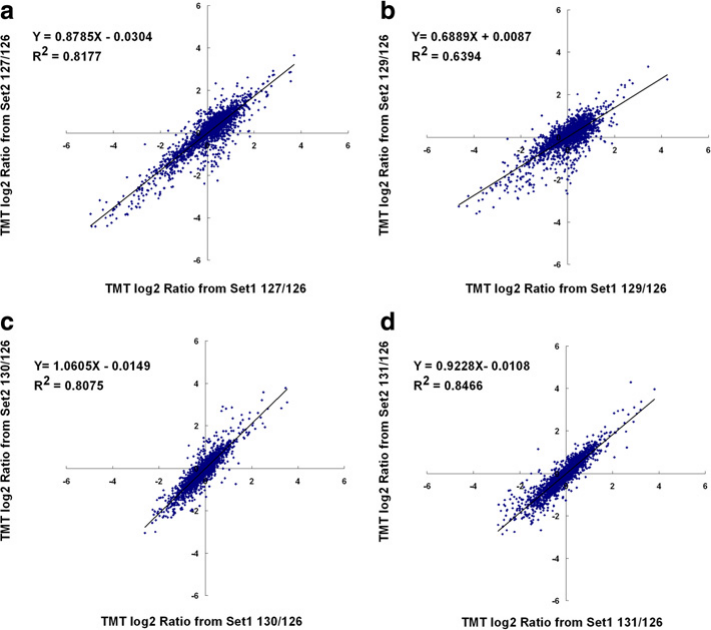
Comparison of log_2_ TMT ratios (127/126,129/126,130/126 and 131/126) for 2290 of quantified proteins which were commonly identified in both sets of biological replicates: set1 and set2. (**a**) log_2_ ratio of 127/126 group (**b**) log_2_ ratio of 129/126 group (**c**) log_2_ ratio of 130/126 group (**d**) log_2_ ratio of 131/126 group. As the internal error for log_2_ ratio of 129/126 group (0.96) was the largest among all these ratios, the correspondence 2-fold was used as a threshold in this study

Total proteins extracted from rice leaves in two sets of biological replicates (set1 and set2) were analyzed by TMT-based shotgun proteomics workflow. A combination of set1 and set2 data showed a total of 4176 proteins were identified with estimated ~ 2% FDR, of which 3206 proteins were commonly identified in both sets (Additional file 1: Table S1). From all the 4176 proteins, 3053 proteins were successfully quantified and about 75% (2290/3053) were commonly quantified in both sets. A variance analysis of the two sets was conducted to determine the quantitative precision. The 2290 proteins identified and quantified in both set1 and set2 were used to assess the quantitative variations from biological and analytical replicates. As shown in panel A-D of Fig. 4, plots of 127/126, 129/126, 130/126 and 131/126 ratios for each quantified proteins between the two sets generated comparable quantification results, as determined by a linear regression analysis that revealed a slope of ≈0.88 at an R^2^ of 0.82 for 127/126, ≈0.69 at an R^2^ of 0.64 for 129/126, ≈1.1 at an R^2^ of 0.81 for 130/126, and ≈0.92 at an R^2^ of 0.85 for 131/126. The results provided a general cross-method validation for the quantitative accuracy of the TMT method in this study. The internal error was defined as the value of the log_2_ TMT ratio at which 95% of all proteins had no deviation from each other, where the deviation was the absolute value of the difference in TMT log_2_ ratios between the x- and y-axis. Thus the internal errors for these four plots at log_2_ scale were 0.92, 0.96, 0.65 and 0.61, which corresponded to 1.9-fold, 2.0-fold, 1.6-fold and 1.5-fold internal variation representing a technical deviation of biological and instrument replicates. The largest deviation ±0.96 at log_2_ 129/126 (corresponding to a ± 2-fold change) was then used as a threshold to identify proteins that were significantly changed in this work.

In the second phase of analysis, we focused on the subsets of quantitative data for differential proteins (both up-regulated and down-regulated proteins) commonly quantified in the biological duplicates at each treatment condition. The RSDs for up-regulated proteins found in RNAi-20 line relative to WT between two sets ranged from 0.02% to 17.4% with average RSD at 6.0% (Additional file 2: Figure S1). The RSD for down-regulated proteins ranged from 0% to 28.7% with average RSD at 10% (Additional file 2: Figure S1), indicating the quantitative proteomics data in this study is reproducible. To further confirm the statistical analysis of the data generated from two sets of biological replicates, we applied another commercially available software package (EasyFit program) for distribution fitting studies with goodness of fit tests for all proteins quantified in each of the individual treatments, in addition to the statistical analysis of the commonly quantified proteins in both sets of biological replicates. The relative expression data for all protein species identified for each of the individual treatments were simultaneously fitted to 50 standard data distribution models using EasyFit (MathWave Technologies, http://www.mathwave.com). Overall, the data were judged to be best fit by the Burr distribution [33] using the Kolmogrov/Sminov, Aderson/Darling and Chi-Squared tests for goodness of fit [34]. The data were found to be normally distributed, as indicated in Additional file 3: Figure S2, Additional file 4: Figure S3, Additional file 5: Figure S4 and Additional file 6: Figure S5), which shows graphs of the probability density function, the cumulative distribution function and both the P-P and Q-Q plots of each of the data sets. Almost identical distribution was found for both sets of biological replicate data, confirming that the relative expression data is statistically reproducible in the two sets of biological replicates (Additional files 3, 4, 5 and 6).

**Fig. 5:**
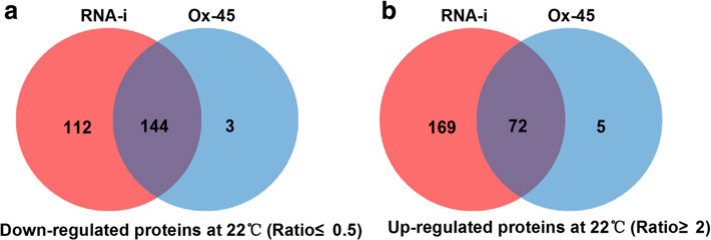
Venn diagram for assessment of differently expressed proteins identified in both *OsNOA1*-silenced and overexpressed lines. The significantly down-regulated proteins were shown in panel (**a**) and up-regulated proteins were shown in panel (**b**)

Our quantitative proteomics data revealed OsNOA1 was decreased by > 2.1-fold (0.46 ± 0.03) in RNAi-20 and increased by > 10-fold (10.40 ± 0.34) in Ox-45 at 22 °C (Additional file 7: Table S2). This observation was also validated by Western blot analysis (see Fig. 1d inset). 256 and 147 proteins were significantly suppressed (≤0.5-fold) in the silenced and Ox-45 lines at 22 °C, respectively (Additional file 7: Table S2 and Fig. 5a). Interestingly, 144 out of the 147 suppressed proteins (98%) in the Ox line were found to be overlapped with the suppressed proteins identified in the silenced line (Fig. 5a). Meanwhile, 241 and 77 proteins were found significantly up-regulated (≥2-fold) in the silenced and Ox lines, respectively (Additional file 8: Table S3 and Fig. 5b). Similarly, 72 out of the 77 (94%) up-regulated proteins in the Ox line were also covered by the up-regulated proteins found in the silenced line (Fig. 5b). More significantly, all of the 33 chloroplast-encoded proteins which suppressed in the Ox line were exactly matched to the list of chloroplast-encoded proteins suppressed in the silenced line (Additional file 7: Table S2).

**Fig. 6:**
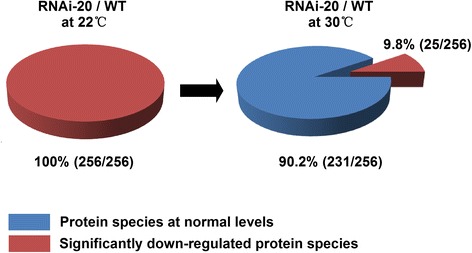
The effects of temperature (22 °C versus 30 °C) on proteome changes. Significantly down-regulated proteins in the RNAi-20 line at 22 °C were compared to those proteins quantified at 30 °C. Red regions represented significantly down-regulated protein species, while blue ones represented protein species at normal levels

In our experimental design, the effects of temperature (22 °C versus 30 °C) on proteome changes could be determined in *OsNOA1*-silenced line over WT. Strikingly, 90% of the suppressed proteins (231/256) were at normal levels as compared to WT at a higher temperature (30 °C) (Fig. 6). The rest 25 proteins including 23 chloroplast ribosomal proteins (out of total 47 identified) and two Rubisco subunits, remained being significantly suppressed (Additional file 7: Table S2). Consistent with the physiological results for similar phenotypes found between the *NOA1*-silenced and Ox lines, our proteomics data showed very comparable protein profiling found in both types of transgenic lines regardless of *OsNOA1* being either down-regulated or highly overexpressed. The proteomics data also revealed the chloroplast ribosomes appear primarily regulated by OsNOA1 in rice.

### Functional classification of differentially expressed proteins

**Fig. 7:**
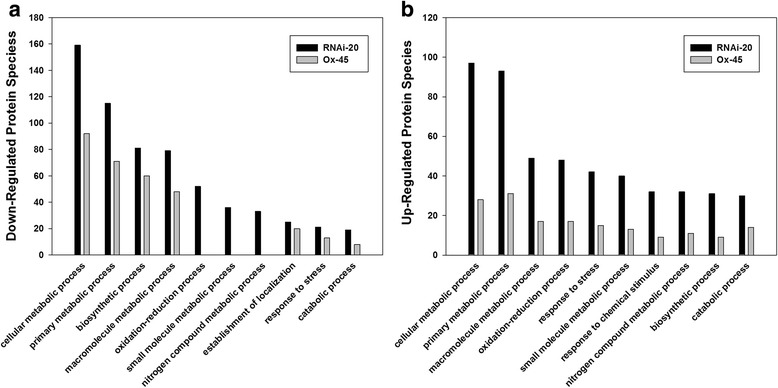
Functional categories of differently expressed proteins in the *OsNOA1*-regulated rice plants: *NOA1*-silenced (RNAi-20) and overexpression (Ox-45). Differentially expressed proteins were classified based on biological process at a GO annotation level of 3 by web-based software Blast2go. The top ten functional categories were selected to cover the data sets for down-regulated (panel **a**) or up-regulated (panel **b**) proteins, respectively

Functional classification of differentially expressed proteins was carried out by web-based software Blast2go. Proteins were classified based on biological process at a GO annotation level of 3, and the top ten functional categories were selected to cover the data sets for up-regulated or down-regulated proteins, respectively (Fig. 7). As expected, the functional categories of differentially expressed proteins in the silenced and Ox-45 lines were also remarkably similar, even though more remarkable changed proteins were identified in the silenced line.

### Subcellular localizations of the differential proteins in both silenced and Ox lines

The subcellular localizations of all the differential proteins in both silenced and Ox lines were predicted using both TargetP (http://www.cbs.dtu.dk/services/TargetP/) and Wolf Psort (http://wolfpsort.org/). It was found that about 90% (229/256) of the suppressed proteins in the silenced line were localized to the chloroplast, including 35 chloroplast-encoded proteins (Additional file 7: Table S2). Similarly, 90% (133/147) of the suppressed proteins in the Ox line were targeted to chloroplast as well, including 33 chloroplast-encoded proteins (Additional file 7: Table S2), which were also identified in the silenced line. Moreover, these chloroplast-encoded proteins were found to be more effectively suppressed in both transgenic lines (Additional file 7: Table S2). The average ratios for the 35 down-regulated, chloroplast-encoded proteins were 0.142, compared to 0.332 for the rest 221 proteins. In contrast, the up-regulated proteins in both transgenic lines were mainly localized to the cytoplasm (33%, 24/72) and mitochondria (26%, 19/72) as indicated in Additional file 8: Table S3.

A further comparison with our previous microarray data from the *NOA1*-silenced line [17] at 22 °C indicated that 73 genes were consistently down-regulated at both the transcriptional and translational levels (Additional file 7: Table S2, Additional file 9: Table S4), accounting for only 32% out of the total 229 suppressed chloroplast-targeted proteins. The comparison results clearly indicated there is a poor expression correlation between levels of transcripts and their cognate proteins in response to *OsNOA1* regulation. Particularly, although the chloroplast ribosomal proteins were effectively suppressed in the silenced line (Additional file 7: Table S2), their transcripts showed little changes regardless of being nuclear-encoded or chloroplast-encoded (Additional file 9: Table S4), suggesting the chloroplast ribosomal proteins are apparently regulated at the translational or post-translational level by OsNOA1.

### Screening for the interaction proteins of OsNOA1

**Fig. 8:**
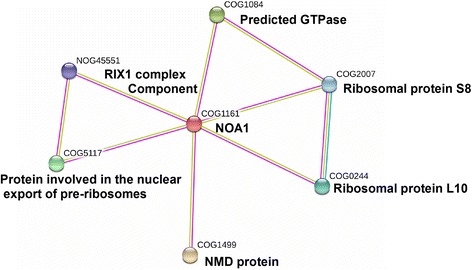
A predicted interaction network of OsNOA1 (COG1161). The interaction network (as displayed by EMBL STRING) for genetically interacting proteins possibly related in function with OsNOA1 was shown. Green lines indicated text mining (literature citations), purple lines indicated high-throughput experimental data (like Y2H), and blue lines indicated database (previous knowledge). Top candidates (score ≥ 0.999) were shown in this figure; of all these proteins, COG0244 and COG2007 were components of ribosomes, others were also involved in the process of translation. NOG45551 and COG1084 took part in the biogenesis of ribosomes, while COG5117 affected the transport of pre-ribosomes. COG1499 affected ribosome stability and mRNA decay

The web-based database for protein-protein interaction network, with known and predicted protein interactions, is a powerful tool to predict possible new protein-protein interactions. Using the EMBL STRING database, we acquired 44 potential interaction partners of OsNOA1 (Additional file 10: Table S5) with highest confidence (score > 0.9) and found about 36% of these proteins (16/44) were ribosomal proteins, many of which were involved in translation. The top six candidates (score = 0.999) were ribosomal protein L10, ribosomal protein S8E, a protein involved in the nucleus export of pre-ribosomes, a NMD protein affecting ribosome stability and mRNA decay, a predicted GTPase, and a component of the RIX1 complex (Fig. 8).

**Fig. 9:**
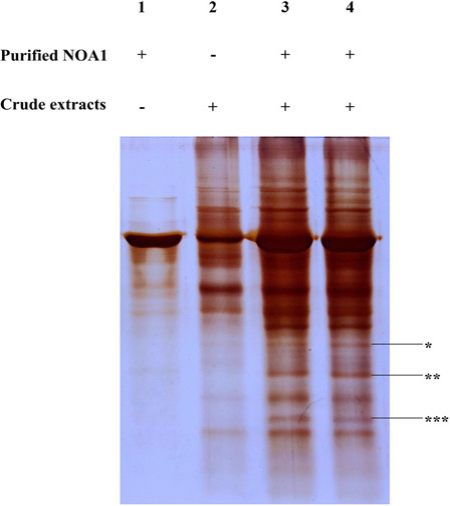
Interaction proteins of OsNOA1 identified by *in vitro* pull-down. The interaction proteins of OsNOA1 were affinity pulled down from crude extracts of rice leaves. Proteins were separated by SDS-PAGE and visualized by silver staining. Lane 1, purified NOA1 only; Lane 2, crude extracts of rice leaves only; Lane 3, pull down reaction replicate 1; Lane4, pull down reaction replicate 2. Possible bands (marked by asterisks) of interaction proteins were identified by mass spectrometry

To further establish whether the ribosomal proteins were the real interaction partners of OsNOA1, an *in vitro* pull-down assay was conducted. We expressed OsNOA1 protein with a deletion of 99 amino acids at its N-terminus (△99-OsNOA1) in bacterial. As the first 99 amino acids were the N-terminal target sequence of OsNOA1, such a deletion would not affect the normal function of NOA1, which was supported by Moreau’s research [2]. Purified △99-OsNOA1 was then incubated with crude protein extract of rice leaves. Potential interaction proteins of OsNOA1 were pulled down through a Ni-IDA resin column under non-denaturing conditions. Acquired protein complex was separated by SDS-PAGE and specific bands were identified by LC-MS/MS. As it was listed in Fig. 9 and Table 2, several chloroplast ribosomal proteins were identified as the interaction candidates of OsNOA1. This result may prove OsNOA1 interacts with chloroplast ribosomes.

**Table 2:** Ribosomal proteins identified in pull-down assay

Description	Score	Protein mass	Isoelectric point
30S ribosomal protein S2 chloroplastic	980.29	27,205.4	10.44
30S ribosomal protein S3 chloroplastic	870.22	27,729	10.4
30S ribosomal protein S4 chloroplastic	625.4	23,465.17	11.59
30S ribosomal protein S5 chloroplastic	307.53	35,007.65	4.68
30S ribosomal protein S8 chloroplastic	677.91	25,137.27	11.04
Plastid-specific 30S ribosomal protein S1	249.05	34,248.84	8.71
Plastid-specific 30S ribosomal protein S2	896.66	26,778.63	8.68
50S ribosomal protein L15 chloroplastic	675.56	28,088.03	11.28
40S ribosomal protein S2	206.37	30,185.09	10.94
40S ribosomal protein S4	340.15	29,758.07	10.85
40S ribosomal protein S8	611.98	25,027.35	11.09
60S ribosomal protein L13a	626.75	23,829.07	10.95
40S Ribosomal protein S6 RPS6–2	406.94	28,682.69	11.35
40S Ribosomal protein S6e	727.9	28,269.42	11.33

## Discussion

### NOA1 regulates the chloroplast proteins in a threshold -dependent manner in rice at lower temperatures

In this work, we found overexpression of *OsNOA1* caused very similar phenotypes to suppression of *OsNOA1* in rice [17], which displayed temperature-sensitive yellowish at 22 °C and restored to nearly normal phenotypes like WT at 30 °C (Fig. 1 and Fig. 2). Further RT-PCR and western blot analyses not only provided direct confirmation for the anticipated overexpression of the *OsNOA1* gene in those Ox lines, but also demonstrated the extent of chlorosis in the Ox lines was positively correlated with the extent of *OsNOA1* overexpression at 22 °C (except for the slightly overexpressed line Ox-2). Based on these initial observations, we hypothesize that there may be an abundance threshold for OsNOA1 to function properly in regulating chlorophyll and Rubisco synthesis and/or other protein metabolism pathways. This hypothesis prompted us to take quantitative proteomics approach to further investigate the effect of OsNOA1 on the global proteome using both the *OsNOA1* silenced and Ox lines grown at two different temperatures.

As expected, our proteomic data revealed basically similar effects on the global proteome occurred when *OsNOA1* was either down-regulated or highly overexpressed at lower temperatures (Additional file 7: Table S2, Additional file 8: Table S3). The proteomics finding agrees well with our hypothesis regarding the mechanism on the effect of *OsNOA1* overexpression, indicating there is apparently a narrow threshold value for OsNOA1 to exercise its normal functions at lower temperatures (Fig. 1). This was also supported by studies of NOA1 homologs. It was widely reported that knock-down or knock-out of *NOA1* caused yellowish phenotypes [2, 11]. Vardi *et al.* [35] reported when a NOA1 homolog (PtNOA) was overexpressed in diatoms, the transformants displayed some negative effects such as reduced growth, impaired photosynthetic efficiency and reduced ability to adhere to surfaces. From the literature review we also found other genes showed the threshold-dependent properties. Samuel *et al.* [36] reported that changing the expression level of SIPK in tomato by either overexpressing or knocking down would similarly result in ROS-sensitive plants.

Our data also suggested OsNOA1 regulates the biosynthesis of chloroplast proteins, regardless chloroplast-encoded or nuclear-encoded. And 90% of the suppressed proteins (229/256 for silenced line or 133/147 for Ox line) were localized in chloroplasts in both types of transgenic lines (Additional file 7: Table S2). Moreover, the abundance of Rubisco, a representative protein in chloroplasts, was remarkably suppressed at the biosynthetic level in both the silenced and Ox lines (Fig. 1e and Fig. 2). The above results tended to support the conclusion that OsNOA1 regulates the biosynthesis of chloroplast proteins in a threshold-dependent manner at lower temperatures. In future, it would be interesting to explore if there is any correlation between this threshold-dependent regulation model and the potential plant adaptation mechanism to temperature stress.

### NOA1 may directly regulate the normal function of chloroplast ribosomes in rice

Among the 256 suppressed proteins identified in the silenced line at 22 °C, 90% of them (229/256) were localized to the chloroplast, including 47 chloroplast ribosome components. These components contained 17 chloroplast-encoded ribosomal proteins and 30 ribosomal proteins that were nuclear-encoded (Additional file 7: Table S2), and so far more than 50 protein components have been identified for chloroplast ribosomes in plants [37]. We also noticed that 90% of the suppressed proteins at 22 °C were restored to normal levels at 30 °C, and the rest 10% (25/256) that remained significantly suppressed at 30 °C were 23 chloroplast ribosomal proteins and 2 Rubisco subunits (LSU and SSU) (Additional file 7: Table S2). These results suggested a primary effect of OsNOA1 on the chloroplast ribosome and a higher temperature may largely rescue some functions of OsNOA1 missing at lower temperatures.

In some other organisms, OsNOA1 homolog was shown to be associated with ribosome assembly or stability, where the YqeH domain plays a critical role [2]. YqeH domain-containing proteins belong to the Era/Obg subfamily of small GTP-binding proteins, which consists of 4 major types: YlqF (for *B. subtilis*), YjeQ (for *E. coli*), and YawG (for *S. pombe*), YqeH (in *B. subtilis*). It has been reported that interactions occurred between YlqF and ribosomal protein L25 and the 50S ribosome subunits were abnormal in YlqF-depleted *B. subtilis* cells [38, 39]. Daigle *et al.* [40] found *E. coli* YjeQ interacted with the 30S subunit of ribosomes in an *in vitro* system. YqeH is essential for the growth of *B. subtilis*, and it contains an N-terminal zinc-ribbon motif used for RNA binding which is also found in several ribosomal proteins. Further analysis indicated YqeH specifically affected the assembly of 70S ribosome and it was involved in either assembly or stabilization of 30S ribosome in *B. subtilis* [41]. A human homolog, i. e. C4orf14, was recently shown to be able to interact with mitochondrial ribosome subunits [14] or with DAP3 [42], a component of mitochondrial ribosomes. Qi *et al*. [18] reported that AtNOA1 was required for thylakoid protein complex assembly, further suggested its’ role in chloroplast ribosome assembly and translation. Our Bioinformatics analysis supported that OsNOA1 is an important candidate that interacts directly with ribosomal proteins (Additional file 10: Table S5 and Fig. 8). Like mutants involved in ribosome biogenesis, the *OsNOA1* silenced rice and the highly overexpressed line (Ox-45) also accumulated considerable number of 16S rRNA precursor at either 22 °C or 30 °C (Fig. 3). More convincingly, His-tag pull-down assays demonstrated OsNOA1 indeed interacted with chloroplast ribosomal proteins (Table 2). Therefore, we believe proper functional interaction between chloroplast ribosomes and NOA1 are critically required for the normal function of chloroplast ribosomes in plants, particularly at lower temperatures.

The next intriguing question is how a higher temperature alleviates the phenotypes. It has been well documented temperature is important for the formation of ribosomes [43], and ribosome activity increases with the rises of temperature [44]. Many organisms with defects in ribosomes showed temperature-sensitive phenotypes like NOA1 rice; In *E. coli*, mutants of genes involved in the assembly of ribosomes showed cold-sensitive phenotypes [45–47]. An *S. cescerevisiae* mutant of the *Drs* gene, which is responsible for the assembly of ribosomes, showed reduced growth when shifted to non-permissive lower temperatures [48]. Similar phenotypes were also reported for the higher plants. A maize mutant named *hcf7* which is impaired in the translation step of chloroplast ribosomes showed cold sensitive phenotype similar to our transgenic rice plants. At 17 °C, this mutant showed dwarf and yellowish phenotypes, whereas its’ phenotypes were nearly normal at 25 °C [49]. A chloroplast-localized protein NUS1 (N protein-utilization substance 1) is known to play roles in regulation of ribosomal RNAs, and its mutant of rice (*v1*) also showed a cold-sensitive yellowish phenotype [50]. Loss function of ribosomal protein L33 or S15, led to a cold-sensitive phenotype of tobacco [51, 52]. Rice mutant *tcd11* (thermo-sensitive chlorophyll-deficient mutant 11) developed albino phenotype at 20 °C, while displayed normal phenotype at 32 °C. Gene mapping showed that *TCD11* encodes the ribosomal small subunit protein S6 in chloroplasts (RPS6) [53]. There have also been *in vitro* recombinant experiments demonstrating the formation of ribosomes requires higher temperatures which are able to stimulate an auto-assembly of the ribosome without need for assembly co-factors [38]. As various assembly cofactors have been identified *in vivo*, such as helicases, GTPases, chaperones, when these co-factors were available could the ribosome be assembled at lower temperatures [54]. Researchers also believed the translation process is sensitive to temperatures. Environmental cold stress arrests the synthesis of proteins by causing ribosomal pausing or retardation of ribosomal biogenesis and RNA processing [55]. Therefore, under the condition of cold stress, ribosomal mutants showed much more defects than those grown at normal temperatures. Given the fact that OsNOA1 has been proved to be a functional GTPase and interacted directly with ribosomal proteins (Fig. 9), we speculate OsNOA1 may directly serve as an assembly co-factor in the formation of chloroplast ribosome complexes. This could reasonably explain why a higher temperature could alleviate these temperature-sensitive phenotypes. But how OsNOA1 regulates those nuclear-encoded proteins is another intriguing question worthy of further investigation, and answering this question may shed more light on the retrograde (organelle to nucleus) signaling pathway [56, 57].

## Conclusions

Our physiological, biochemical, microarray, proteomics and bioinformatics results collectively show that both *NOA1* silenced and overexpression transgenic lines display similar phenotypes with comparable proteomics profiles and functional classifications. Our results reveal that OsNOA1 functions in a threshold-dependent manner to regulate the biosynthesis of chloroplast proteins in rice at lower temperatures. Strikingly, higher temperatures can effectively complement the NOA1 functions in *OsNOA1*-silenced rice by rescuing 90% of the suppressed proteins to normal expression levels. Furthermore, we confirmed NOA1 is directly interacted with chloroplast ribosomal proteins. These findings strongly suggest NOA1 may directly serve as an assembly co-factor in the formation of chloroplast ribosome complexes. In addition, NOA1 was demonstrated to regulate the chloroplast ribosomal proteins at the translational level for both nuclear-encoded (via plastid retrograde signaling) and chloroplast-encoded proteins, offering a new piece of evidence that NOA1 appears to directly regulate the normal function of chloroplast ribosomes.

## Additional files

Additional file 1: **Table S1.** Complete list of all identified proteins. Complete list of all identified proteins (4176 proteins) were organized by protein accession numbers. (XLS 4120 kb)

Additional file 2: **Figure S1.** Venn diagrams of differentially expressed identified protein species in duplicated sets of biological samples. For comparison of RNAi-20 vs. WT, the maximum RSDs for up-regulated (A) and down-regulated (B) protein species were 17% (A) and 29% (B) with average RSDs = 6% and 10%, respectively. For comparison of Ox-45 vs. WT, the maximum RSDs for up-regulated (C) and down-regulated (D) protein species were 25% and 43% each, with average RSDs = of 9% and 17% respectively. (TIFF 1721 kb)

Additional file 3: **Figure S2.** Comparison of the probability density function of the relative expression data (Treated/WT) for all proteins quantified for each of the individual transgenic line in both sets of biological replicates. Each set of data was simultaneously fit to 50 standard data distribution models using the EasyFit software suit (MathWave Technologies, http://www.mathwave.com). The data were judged to be best fit by the Burr distribution, using the Kolmogrov/Sminov, Aderson/Darling and Chi-Squared tests for goodness of fit. The four top panels (A-D) represent the log2 ratios for 127-tag (RNAi-20 at 22 °C)/126-tag (WT 22 °C), for 129-tag (Ox-45 at 22 °C)/126-tag (WT 22 °C), for 130-tag (WT 30 °C)/126-tag (WT 22 °C), and for 131-tag (RNAi-20 30 °C)/126-tag (WT 22 °C), respectively, in biological set 1. The bottom four panels (E-H) represent the equivalent of A-D, but for biological set 2. Each data set was found to be consistent and normally distributed, as shown in this graph. (TIFF 482 kb)

Additional file 4: **Figure S3.** Comparison of the cumulative distribution function (CDF) of the relative expression data (Treated/WT) for all proteins quantified for each of the individual treatments in both sets of biological replicates. The top four panels (A-D) represent the log2 ratios for 127-tag (RNAi-20 at 22 °C)/126-tag (WT 22 °C), for 129-tag (Ox-45 at 22 °C)/126-tag (WT 22 °C), for 130-tag (WT 30 °C)/126-tag (WT 22 °C), and for 131-tag (RNAi-20 30 °C)/126-tag (WT 22 °C) in biological set 1. The bottom four panels (E-H) represent the equivalent of A-D, but for biological set 2. Consistent distribution for each data set between the two sets of biological replicates was demonstrated. (TIFF 336 kb)

Additional file 5: **Figure S4.** Comparison of the probability-probability (P-P) plot of the same data sets in both sets of biological replicates as described in Additional file 2: **Figure S1.** The top four panels (A-D) represent the log2 ratios for 127/126, 129/126, 130/126 and 131/126 respectively in biological set 1. The bottom four panels (E-H) represent the equivalent of A-D but for biological set 2. The P-P plot is a graph of the empirical CDF values plotted against the theoretical CDF values, and used to determine how well a specific distribution fits to the observed data. The approximately linear plots for each of the data sets confirmed the correct theoretical distribution model and data consistency between two sets of biological replicates. (TIFF 339 kb)

Additional file 6: **Figure S5.** Comparison of the quantile-quantile (Q-Q) plot of the same data sets in both sets of biological replicates as described in Additional file 2: **Figure S1.** The top four panels (A-D) represent the log2 ratios for 127/126, 129/126, 130/126 and 131/126 respectively in biological set 1. The bottom four panels (E-H) represent the equivalent of A-D but for biological set 2. The Q-Q plot is a graph of the input (observed) data values plotted against the theoretical (fitted) distribution quantiles. The approximately linear plots for each of the data sets confirm the data consistency between two sets of biological replicates. (TIFF 314 kb)

Additional file 7: **Table S2.** Down-regulated protein species in the silenced line at 22 °C and corresponding ratios at 30 °C. A total of 256 down-regulated proteins in the silenced line at 22 °C were organized by localization and functional groups. Ratios of these proteins in silenced line grown at 30 °C as well as corresponding ratios in line Ox-45 grown at 22 °C were also included in the table. (XLS 90 kb)

Additional file 8: **Table S3.** Up-regulated protein species in the silenced line at 22 °C and corresponding ratios at 30 °C. A total of 241 up-regulated proteins in the silenced line at 22 °C were organized by functional groups. Corresponding ratios of these proteins in line Ox-45 were also included in the table. (XLS 65 kb)

Additional file 9: **Table S4.** A comparison of proteomics data with previously published microarray data. Transcripts matched to down-regulated proteins in the silenced line at 22 °C were listed in this table (mentioned in Table S2). (XLS 86 kb)

Additional file 10: **Table S5.** Predicted functional partners of OsNOA1. 44 potential interaction partners of OsNOA1 predicted by STRING database were listed in this table. (XLS 22 kb)

## Abbreviations

COG: Clusters of orthologous groups of proteins
DDA: Data-dependent acquisition
FA: Formic acid
FDR: False discovery rate
GO: Gene ontology
HCD: High energy collision dissociation
HpRP: High pH reverse phase
NOA1: NO-associated protein 1
RNAi: RNA interference
RSD: Relative standard deviation
SCX: Strong cation exchange
SPE: Solid-phase extraction
TCEP: Tris-(2-carboxyethyl) phosphine
TMT: Tandem mass tag
