# Supplementary figures and images for: OsNOA1 functions in a threshold-dependent manner to regulate chloroplast proteins in rice at lower temperatures
Source: BMC Plant Biol. 2018 Mar 16;18:44. doi: 10.1186/s12870-018-1258-9 (PMC5857130; doi:10.1186/s12870-018-1258-9)

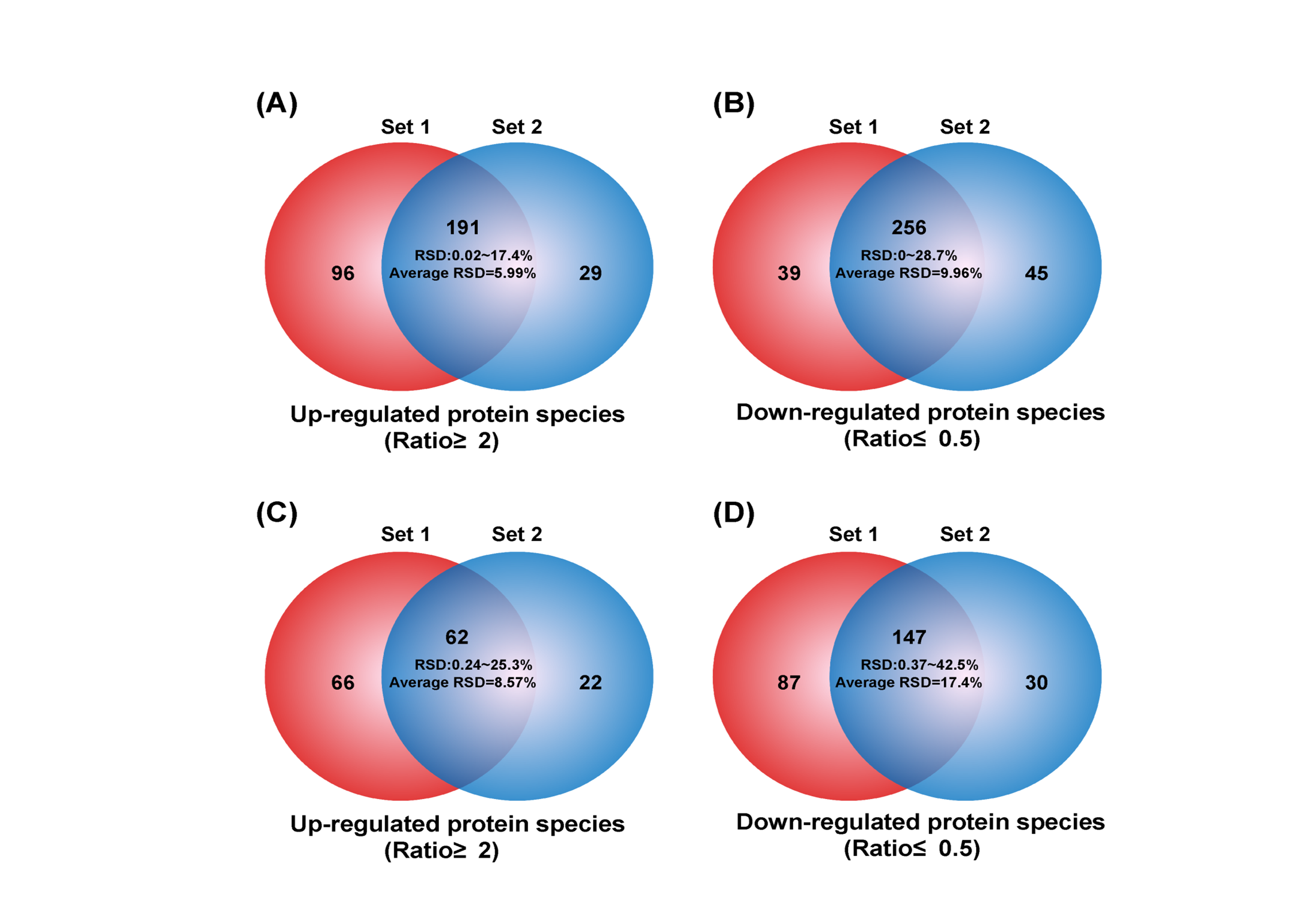

Supplement: Supplementary file 2 — Figure S1. Venn diagrams of differentially expressed identified protein species in duplicated sets of biological samples. For comparison of RNAi-20 vs. WT, the maximum RSDs for up-regulated (A) and down-regulated (B) protein species were 17% (A) and 29% (B) with average RSDs = 6% and 10%, respectively. For comparison of Ox-45 vs. WT, the maximum RSDs for up-regulated (C) and down-regulated (D) protein species were 25% and 43% each, with average RSDs = of 9% and 17% respectively. (TIFF 1721 kb) [file 12870_2018_1258_MOESM2_ESM.tif]

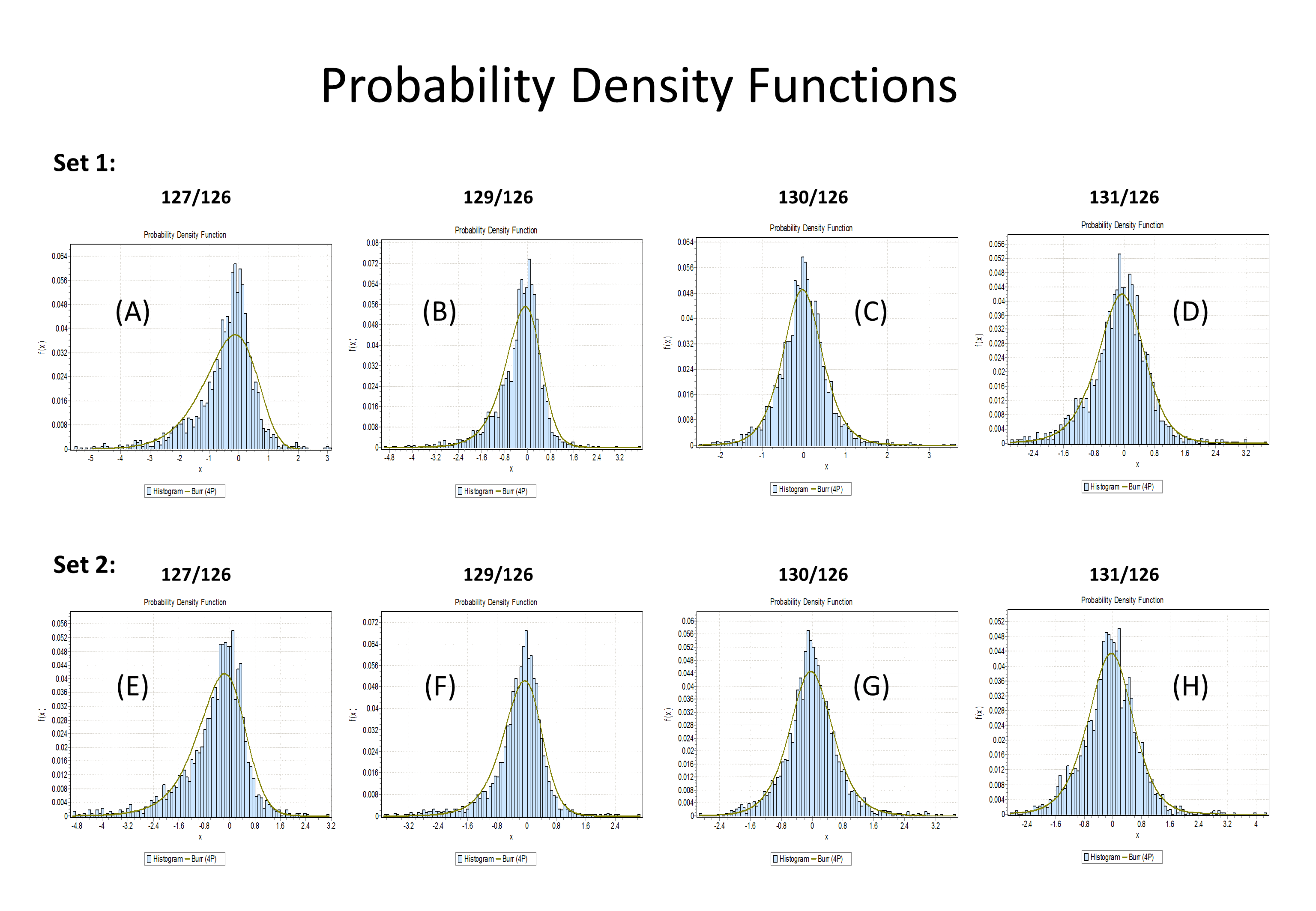

Supplement: Supplementary file 3 — Figure S2. Comparison of the probability density function of the relative expression data (Treated/WT) for all proteins quantified for each of the individual transgenic line in both sets of biological replicates. Each set of data was simultaneously fit to 50 standard data distribution models using the EasyFit software suit (MathWave Technologies, http://www.mathwave.com). The data were judged to be best fit by the Burr distribution, using the Kolmogrov/Sminov, Aderson/Darling and Chi-Squared tests for goodness of fit. The four top panels (A-D) represent the log2 ratios for 127-tag (RNAi-20 at 22 °C)/126-tag (WT 22 °C), for 129-tag (Ox-45 at 22 °C)/126-tag (WT 22 °C), for 130-tag (WT 30 °C)/126-tag (WT 22 °C), and for 131-tag (RNAi-20 30 °C)/126-tag (WT 22 °C), respectively, in biological set 1. The bottom four panels (E-H) represent the equivalent of A-D, but for biological set 2. Each data set was found to be consistent and normally distributed, as shown in this graph. (TIFF 482 kb) [file 12870_2018_1258_MOESM3_ESM.tif]

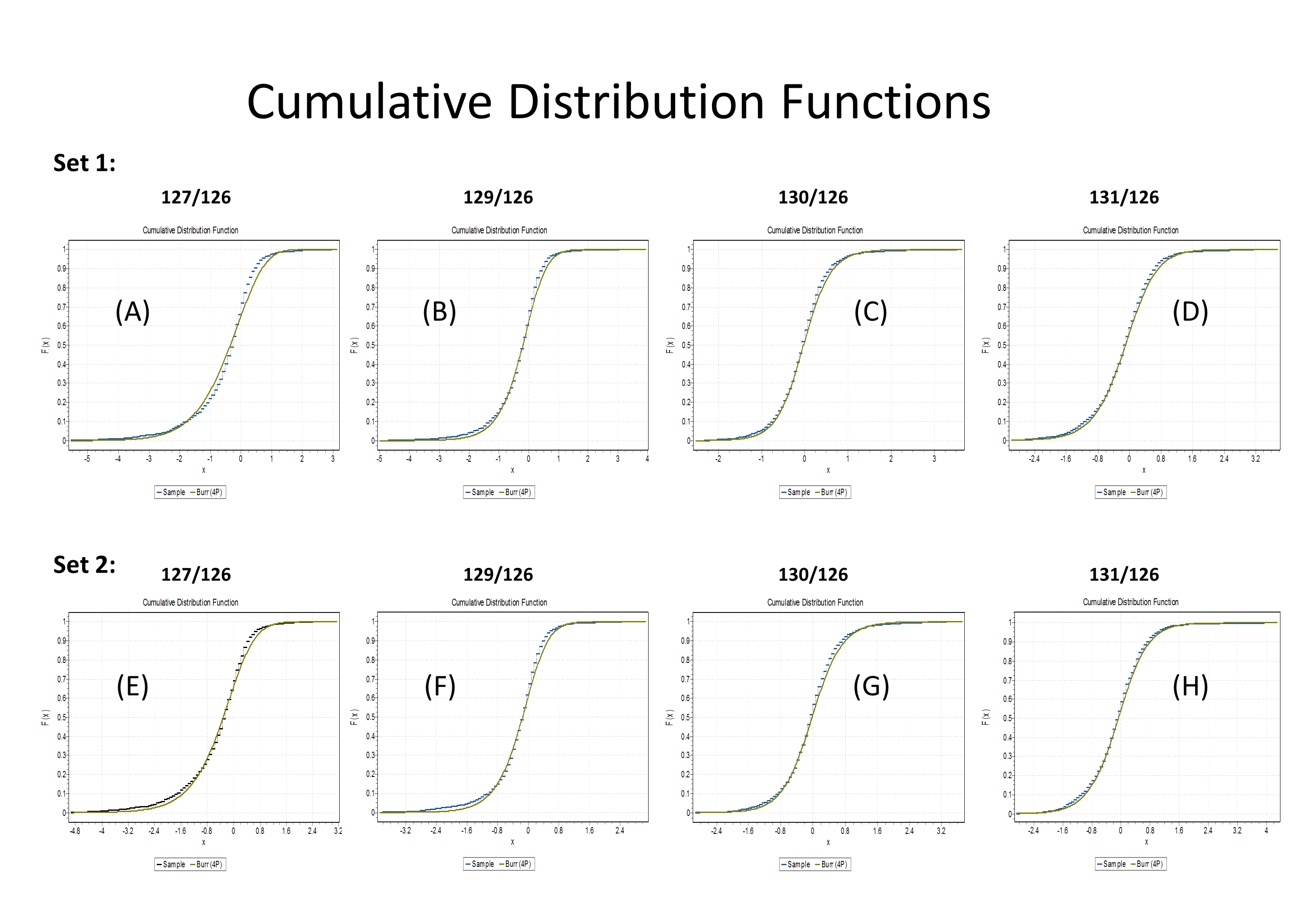

Supplement: Supplementary file 4 — Figure S3. Comparison of the cumulative distribution function (CDF) of the relative expression data (Treated/WT) for all proteins quantified for each of the individual treatments in both sets of biological replicates. The top four panels (A-D) represent the log2 ratios for 127-tag (RNAi-20 at 22 °C)/126-tag (WT 22 °C), for 129-tag (Ox-45 at 22 °C)/126-tag (WT 22 °C), for 130-tag (WT 30 °C)/126-tag (WT 22 °C), and for 131-tag (RNAi-20 30 °C)/126-tag (WT 22 °C) in biological set 1. The bottom four panels (E-H) represent the equivalent of A-D, but for biological set 2. Consistent distribution for each data set between the two sets of biological replicates was demonstrated. (TIFF 336 kb) [file 12870_2018_1258_MOESM4_ESM.tif]

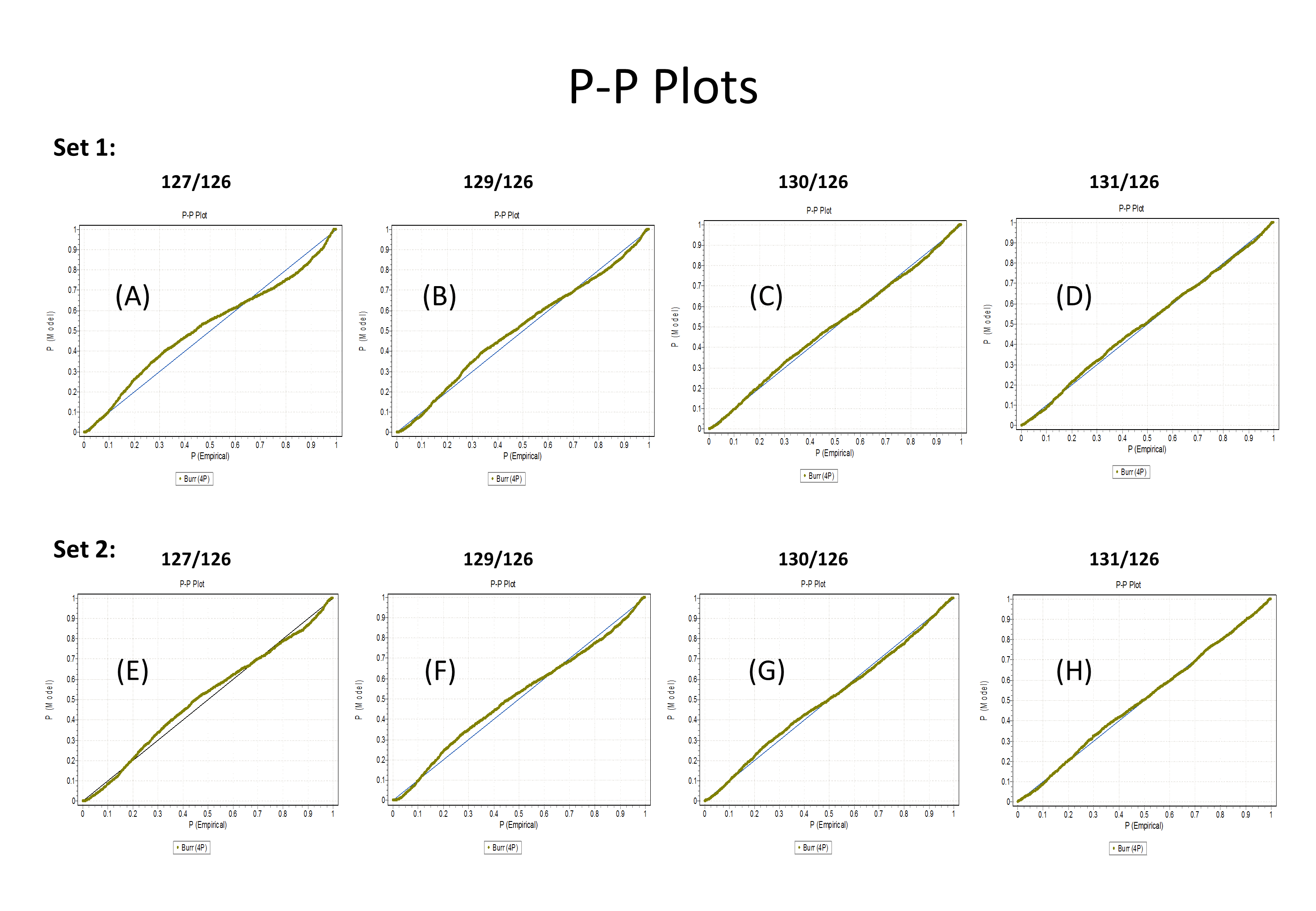

Supplement: Supplementary file 5 — Figure S4. Comparison of the probability-probability (P-P) plot of the same data sets in both sets of biological replicates as described in Additional file 2: Figure S1. The top four panels (A-D) represent the log2 ratios for 127/126, 129/126, 130/126 and 131/126 respectively in biological set 1. The bottom four panels (E-H) represent the equivalent of A-D but for biological set 2. The P-P plot is a graph of the empirical CDF values plotted against the theoretical CDF values, and used to determine how well a specific distribution fits to the observed data. The approximately linear plots for each of the data sets confirmed the correct theoretical distribution model and data consistency between two sets of biological replicates. (TIFF 339 kb) [file 12870_2018_1258_MOESM5_ESM.tif]

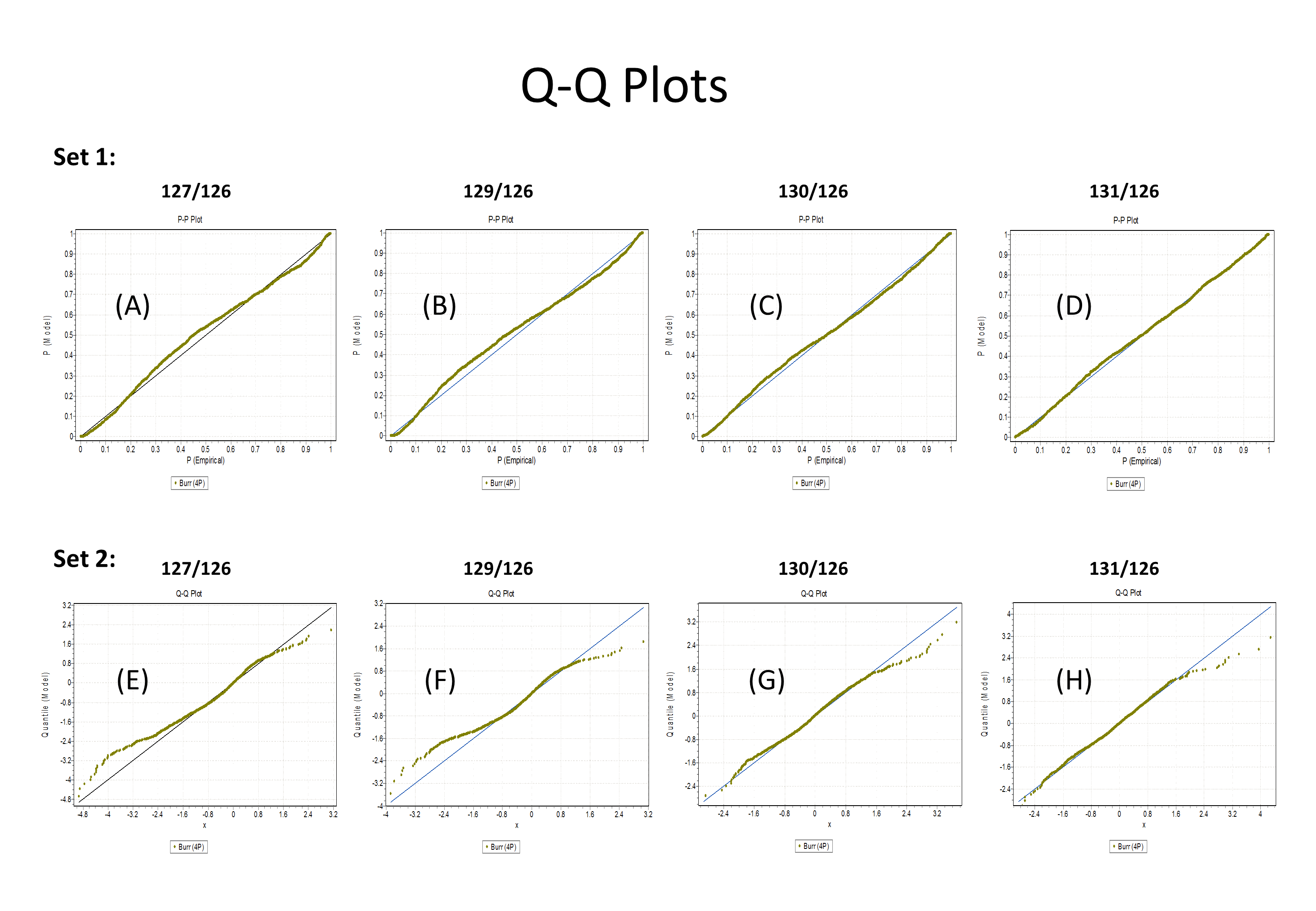

Supplement: Supplementary file 6 — Figure S5. Comparison of the quantile-quantile (Q-Q) plot of the same data sets in both sets of biological replicates as described in Additional file 2: Figure S1. The top four panels (A-D) represent the log2 ratios for 127/126, 129/126, 130/126 and 131/126 respectively in biological set 1. The bottom four panels (E-H) represent the equivalent of A-D but for biological set 2. The Q-Q plot is a graph of the input (observed) data values plotted against the theoretical (fitted) distribution quantiles. The approximately linear plots for each of the data sets confirm the data consistency between two sets of biological replicates. (TIFF 314 kb) [file 12870_2018_1258_MOESM6_ESM.tif]
